# Supplementary material for: An Aggrephagy-Related LncRNA Signature for the Prognosis of Pancreatic Adenocarcinoma
Source: Genes (Basel). 2023 Jan 2;14(1):124. doi: 10.3390/genes14010124 (PMC9859148; doi:10.3390/genes14010124)
Supplement: Supplementary file 1 [file genes-14-00124-s001.zip › FigureS1.pdf]

LncRNA ID	Hazard ratio	HR.95L	HR.95H	pvalue
AC025048.4	0.331	0.144	0.764	0.010
AL008729.2	0.674	0.502	0.905	0.009
AC138356.1	0.115	0.024	0.541	0.006
AL034374.1	0.365	0.140	0.953	0.039
AC015660.1	1.449	1.033	2.032	0.032
AC013400.1	0.285	0.095	0.851	0.025
MED14OS	0.354	0.173	0.727	0.005
CASC8	1.833	1.360	2.470	<0.001
NFIA-AS2	0.209	0.055	0.793	0.021
AP000894.4	0.696	0.486	0.996	0.048
MEG3	0.760	0.592	0.975	0.031
CH17-340M24.3	0.480	0.283	0.812	0.006
AC005332.4	0.615	0.396	0.953	0.030
LRRC8C-DT	0.219	0.055	0.877	0.032
AC022098.1	0.380	0.154	0.935	0.035
SUGT1P4-STRA6LP	0.130	0.032	0.526	0.004
AC025165.1	0.240	0.066	0.879	0.031
LINC02593	0.606	0.430	0.855	0.004
AC009019.1	0.193	0.038	0.980	0.047
ZMIZ1-AS1	0.515	0.272	0.975	0.042
AC068228.1	1.725	1.014	2.936	0.044
AC007292.1	0.482	0.251	0.923	0.028
AC078923.1	2.179	1.270	3.738	0.005
AC080013.4	0.416	0.183	0.948	0.037
FGF14-AS2	0.717	0.525	0.980	0.037
CACNA1G-AS1	0.113	0.016	0.797	0.029
NCAM1-AS1	0.029	0.001	0.916	0.044
ZNF236-DT	0.382	0.189	0.774	0.008
AC087501.4	0.338	0.141	0.811	0.015
MEG9	0.401	0.176	0.911	0.029
AC002059.1	0.529	0.309	0.903	0.020
LINC01160	0.352	0.142	0.873	0.024
AC104117.5	0.283	0.085	0.938	0.039
AL139274.2	0.172	0.031	0.967	0.046
AF111169.3	0.444	0.234	0.841	0.013
CCND2-AS1	0.258	0.083	0.802	0.019
ZNF710-AS1	0.579	0.357	0.938	0.026
AP000255.1	0.397	0.187	0.843	0.016
AC005062.1	0.443	0.201	0.979	0.044
AC004921.1	0.584	0.352	0.969	0.037
LINC01091	0.400	0.185	0.862	0.019
FAM83A-AS1	1.315	1.064	1.626	0.011
AC004540.2	0.490	0.273	0.881	0.017

Z97832.2	0.322	0.146	0.708	0.005
PAN3-AS1	0.367	0.222	0.605	<0.001
LINC02245	0.177	0.058	0.538	0.002
LINC02600	0.413	0.221	0.773	0.006
AC009159.3	0.472	0.295	0.757	0.002
AC090114.2	0.472	0.285	0.781	0.003
AC110995.1	0.508	0.279	0.925	0.027
SRGAP3-AS4	0.525	0.281	0.983	0.044
SEMA6A-AS2	0.122	0.016	0.942	0.044
AC092171.5	0.555	0.361	0.854	0.007
C9orf139	0.209	0.068	0.640	0.006
INTS6L-AS1	0.304	0.098	0.938	0.038
AC024270.4	0.088	0.012	0.653	0.017
ZNF667-AS1	0.595	0.364	0.972	0.038
