# Supplementary material for: An Aggrephagy-Related LncRNA Signature for the Prognosis of Pancreatic Adenocarcinoma
Source: Genes (Basel). 2023 Jan 2;14(1):124. doi: 10.3390/genes14010124 (PMC9859148; doi:10.3390/genes14010124)
Supplement: Supplementary file 1 [file genes-14-00124-s001.zip › Supplementary Figures.docx]

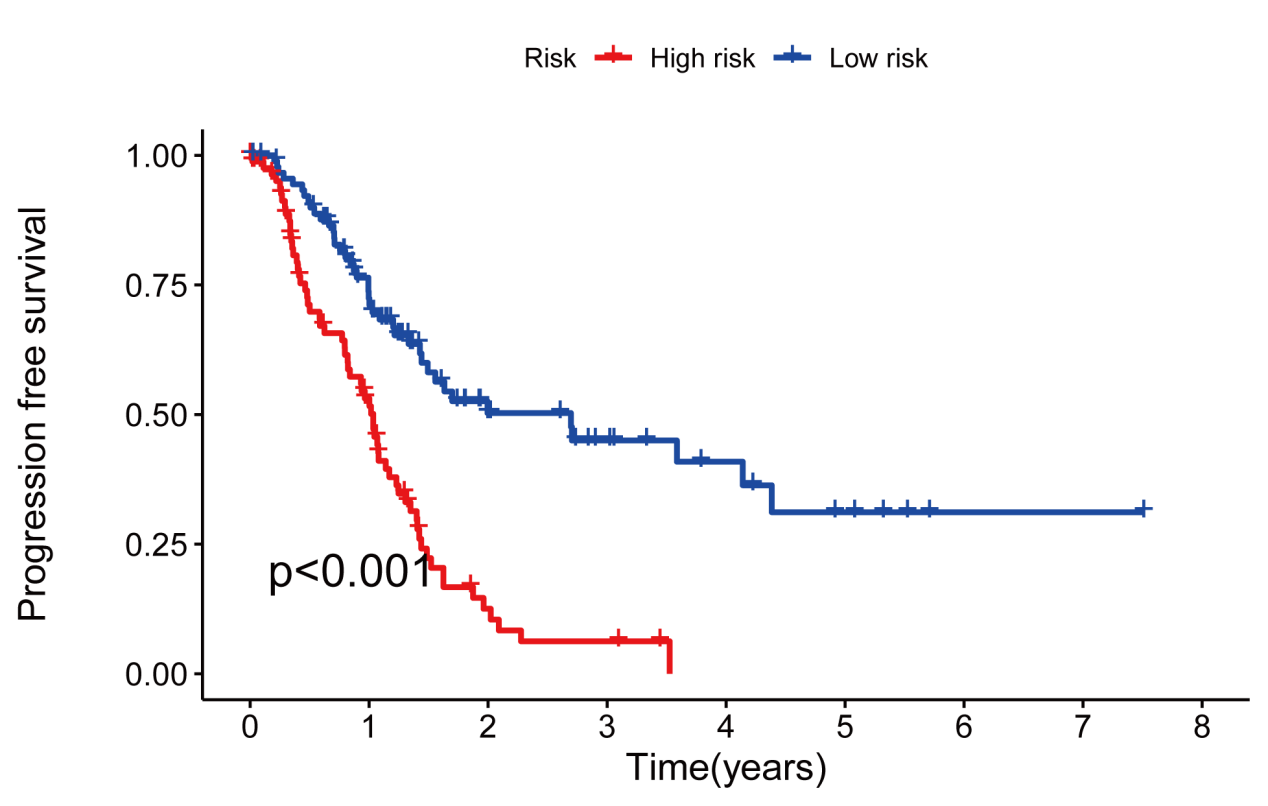


Supplementary Figure2: the progression-free survival (PFS) of the total dataset.


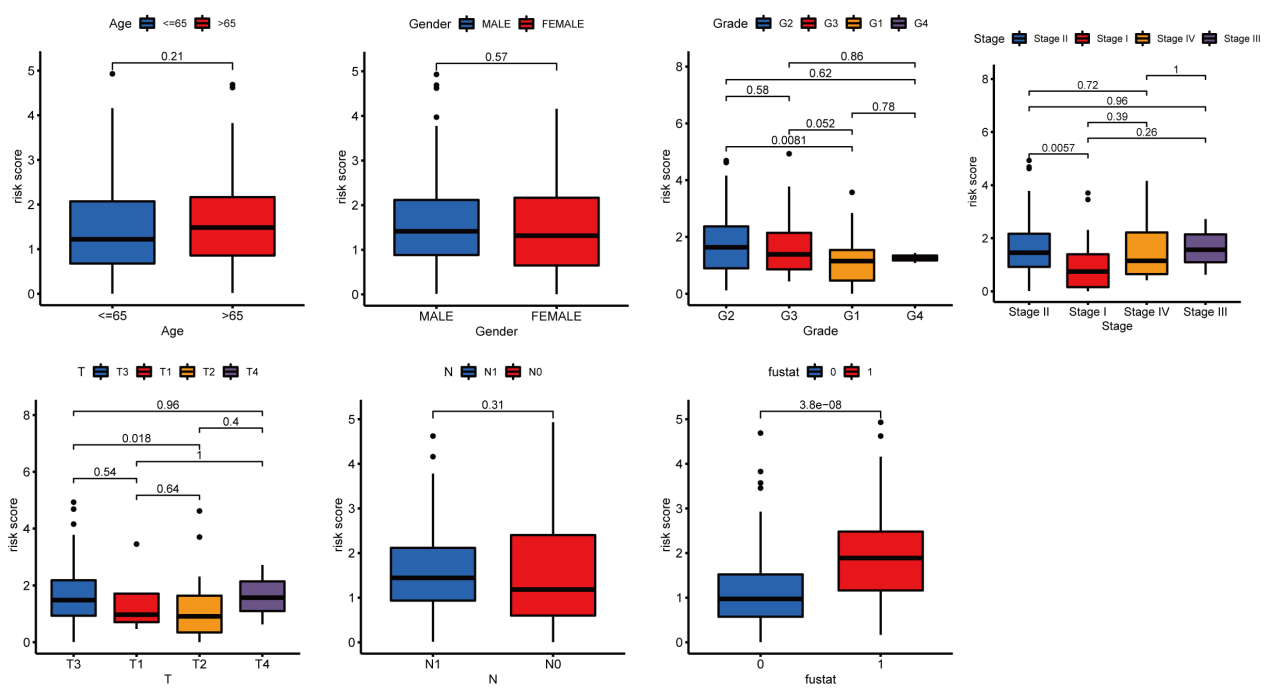


Supplementary Figure 3: Differences in clinicopathological characteristics between high and low risk groups(Age,Gender,Grade,Stage,T,N,Fustat).


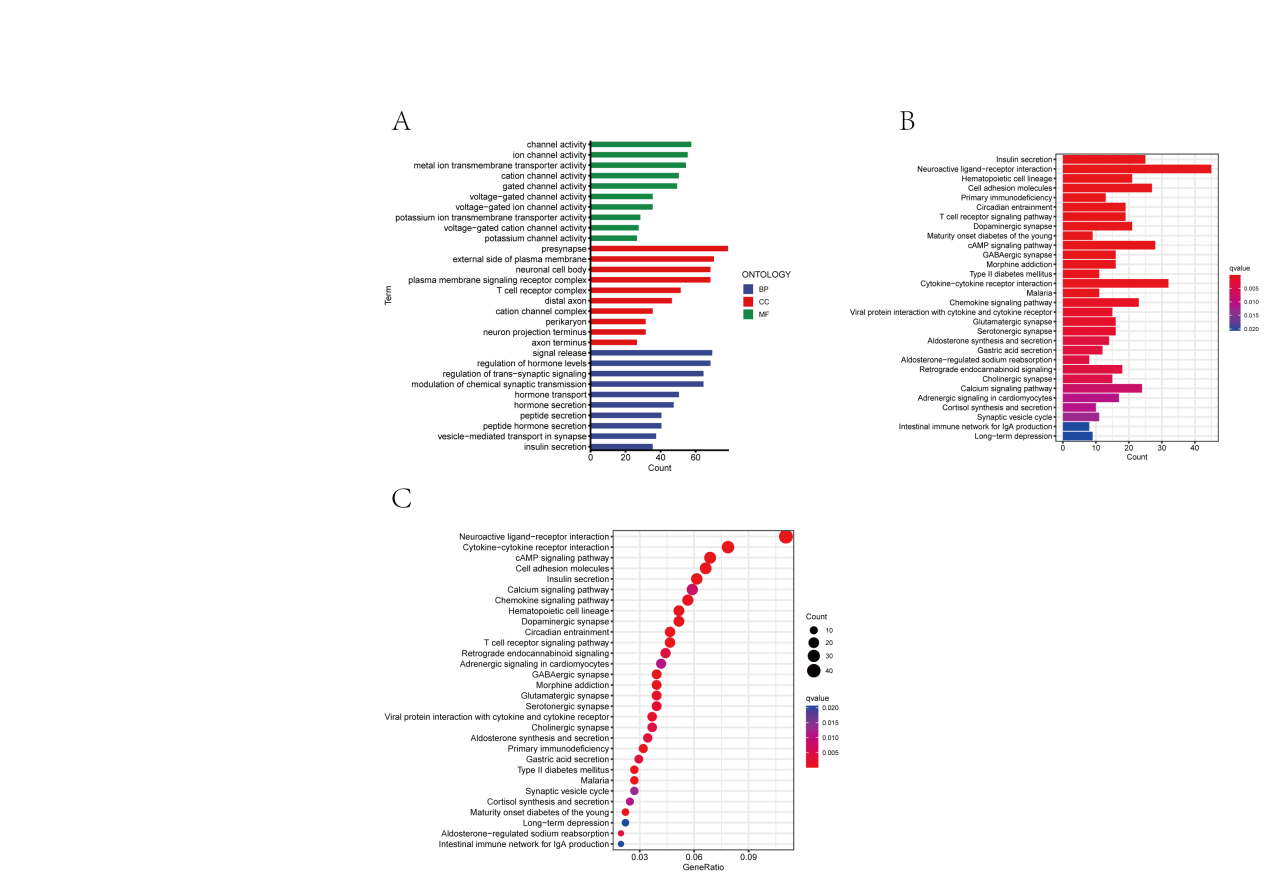


Supplementary Figure 4: Results of functional enrichment.
