# Supplementary figures and images for: An Aggrephagy-Related LncRNA Signature for the Prognosis of Pancreatic Adenocarcinoma
Source: Genes (Basel). 2023 Jan 2;14(1):124. doi: 10.3390/genes14010124 (PMC9859148; doi:10.3390/genes14010124)

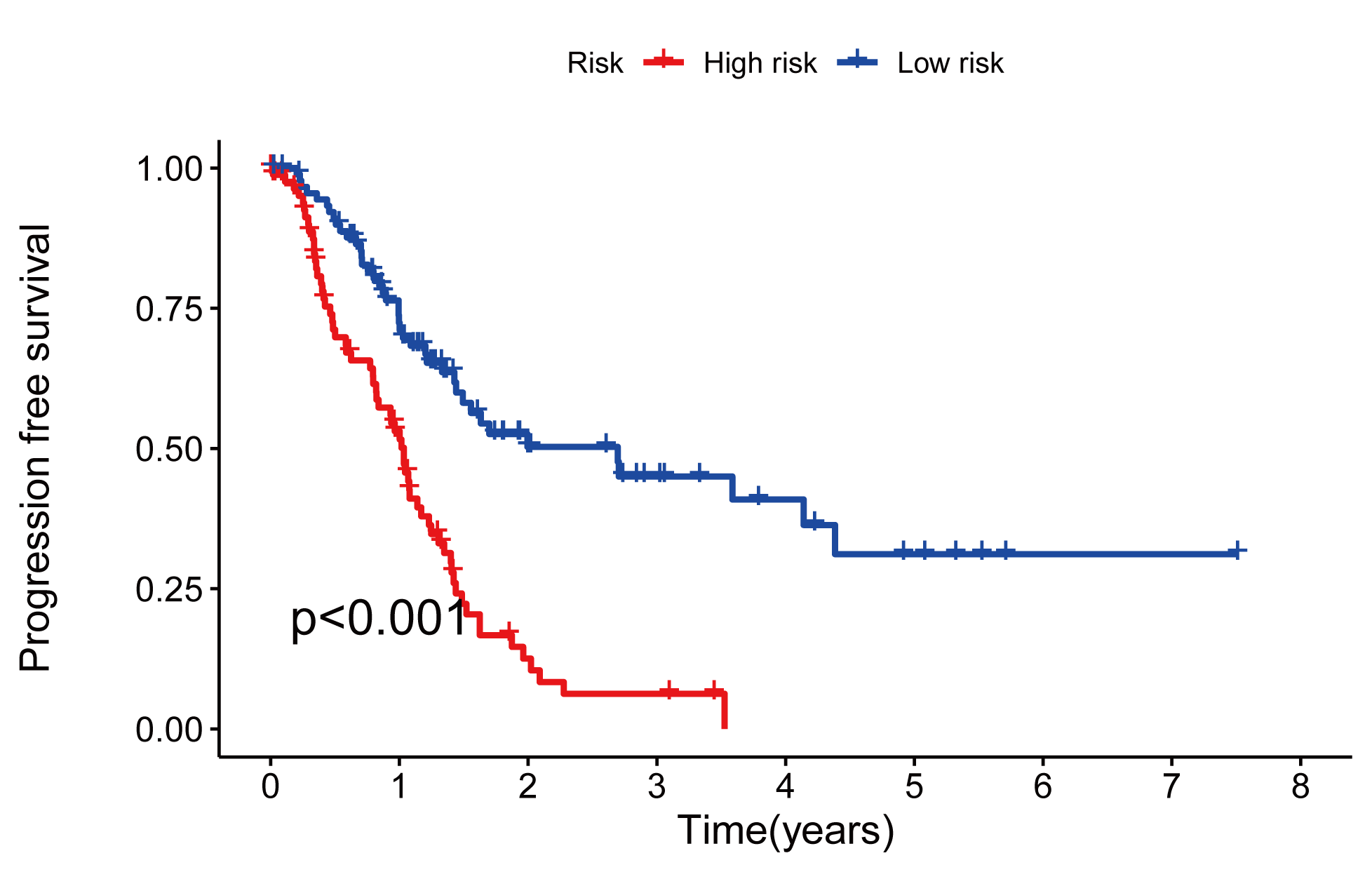

Supplement: Supplementary file 1 [file genes-14-00124-s001.zip › FigureS2.tif]

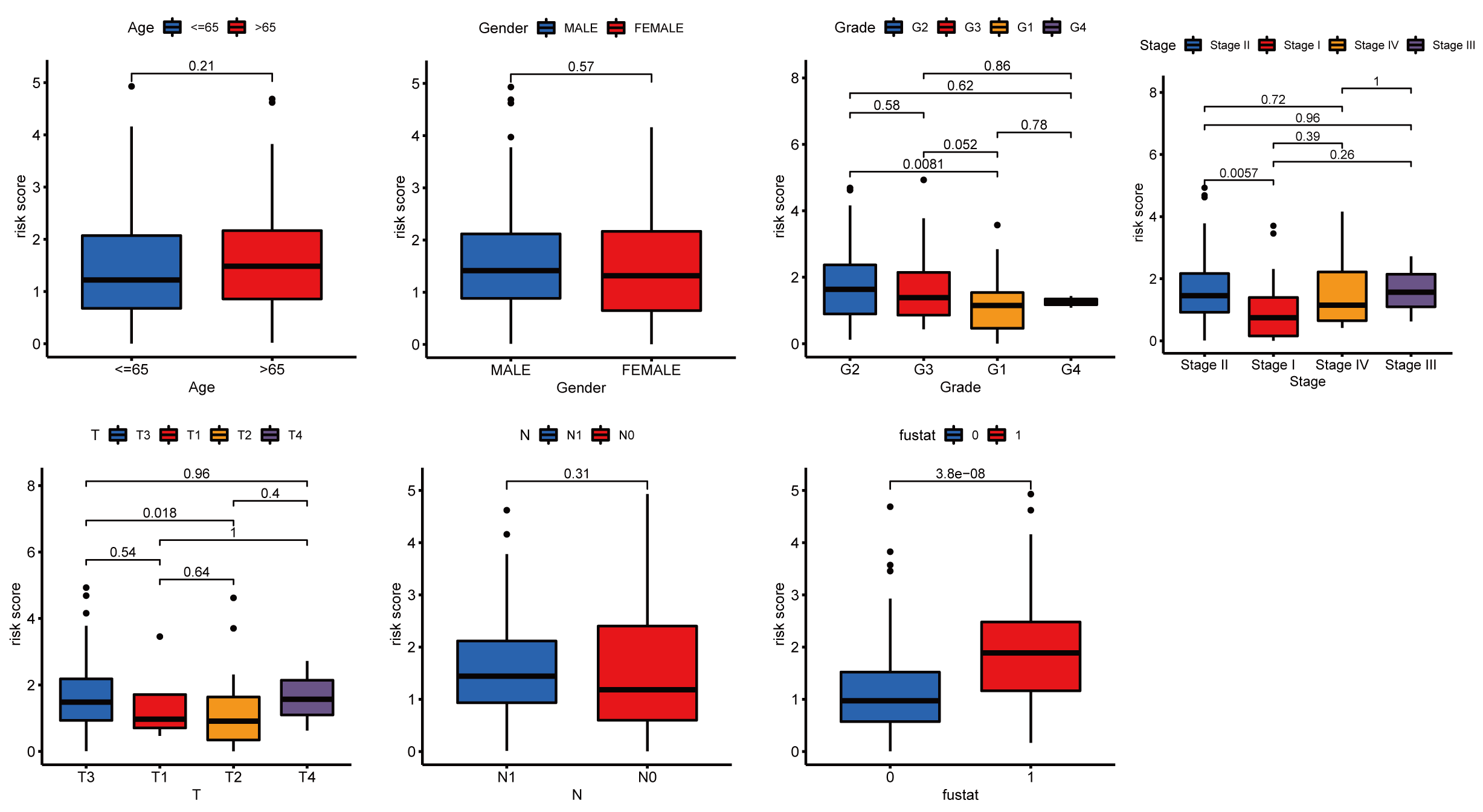

Supplement: Supplementary file 1 [file genes-14-00124-s001.zip › FigureS3.tif]

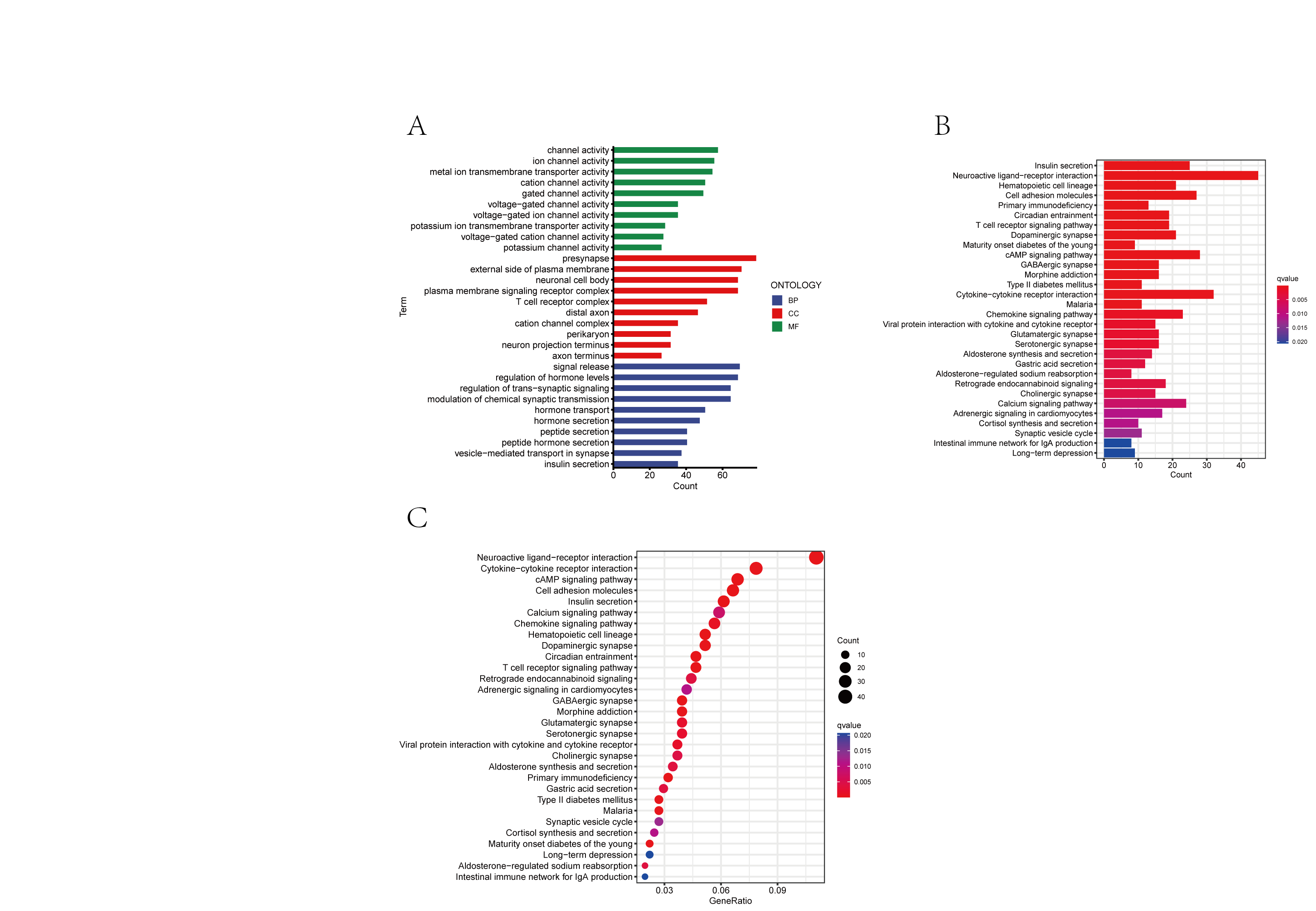

Supplement: Supplementary file 1 [file genes-14-00124-s001.zip › FigureS4.tif]
